# Effects of season, age and parasite management practices on gastro – intestinal parasites in pigs kept outdoors in Ireland

**DOI:** 10.1186/s13620-025-00297-0

**Published:** 2025-05-04

**Authors:** Nipuna Sahan Senanayake, Laura Boyle, Keelin O’Driscoll, Ophélie Menant, Fidelma Butler

**Affiliations:** 1 Pig Development Department, Teagasc Animal and Grassland Research and Innovation Centre, Fermoy, Co, Cork, Ireland; 2 School of Biological, Earth and Environmental Sciences, University College Cork, Cork, Ireland

**Keywords:** Faecal egg count, Anthelmintic, Paddock rotation, Protozoa, Helminths, Strongyles

## Abstract

Outdoor farming offers pigs considerable behavioural freedom and better consumer acceptance than intensive, indoor systems. However, gastro – intestinal (GI) parasites pose a significant health and welfare challenge for pigs reared outdoors. The aim of this study was to ascertain effects of management, season and animal factors such as age, on a range of different GI parasites in Irish pigs farmed outdoors. Sixty-five pig faecal samples (a mix from at least 2–4 animals per paddock) were collected from 65 paddocks across 20 outdoor pig farms, over two visits (1^st^ visit – February/May-December 2023, *n* = 37, 2^nd^ visit– July/October 2023, *n* = 28). Samples were collected and mixed thoroughly to achieve a paddock level sample. Data were also collected related to pig characteristics (grower/fatteners or sows and boars), anthelmintic usage (Yes/No) and paddock rotation (Yes/No) and categorized at paddock level. Samples were analysed using the McMaster floatation method, faecal egg count (FEC) was calculated, and GI parasites were identified by morphology. Generalized linear mixed models were used to analyse the effect of season, age, anthelmintic usage and paddock rotation on FEC. Four parasite taxa were identified (*Eimeria/Isospora* spp., strongyles, *Ascaris suum* and *Trichuris suis*). Infection rates were > 80% for *Eimeria/Isospora* spp. and strongyles, 31% for *A. suum* and 9% for *T. suis* for both visits. *Eimeria/Isospora* spp. FEC was higher at the 2^nd^ visit (*P* < 0.001) and strongyles FEC was higher at the 1^st^ visit (*P* < 0.05). Fattener pigs had higher FEC for *Eimeria/Isospora* spp. (*P* < 0.01) and sows/boars had higher strongyle counts (*P* < 0.05). Strongyle count was lower with anthelmintic use (*P* < 0.05) and *Eimeria/Isospora* spp. count was lower (*P* = 0.05) with paddock rotation when anthelmintics were used. Lower winter temperatures may have influenced the seasonal variation in strongyle FEC. This study provides a comprehensive picture of GI parasites in outdoor pig farms in Ireland in terms of the taxa, their prevalence and risk factors.

## Introduction

Infection with gastro–intestinal (GI) parasites is a cause of ongoing and increasing concern in livestock production because of detrimental economic and animal welfare effects, which negatively impact sustainability of the industry [1–3]. Detrimental implications for animal health are associated with morbidity, reduced feed conversion efficiency and growth, as well as treatment costs [4, 5]. Effective control of GI parasites requires a combined use of anti-parasitic drugs and management practices such as rotational grazing [6]. Increasingly there are also concerns for resistance to treatment [7]. GI parasites, particularly helminths, affect domestic pigs in all production systems around the world [8]. In pigs, infections impair intestinal absorption, prolong the fattening period, delay or hamper immunity after vaccination, reduce meat quality, and in the case of young pigs, may cause death, due to diarrhoea and dehydration [9, 10]. In spite of the potential clinical implications, parasitic infections in pigs seldom cause clinical symptoms, especially in the case of helminths, and diagnosis is generally based on laboratory examination [8, 10]. The subclinical nature of helminth parasitic infection means that they receive less attention compared to other parasites in other livestock species. An additional risk of GI infections in pigs is the potential for zoonoses associated with helminth species such as *Ascaris* and *Trichuris* [11].

While the majority of pigs in developed countries are farmed intensively indoors, the increasing interest in welfare-friendly meat means that there is growth in the outdoor pig production sector [12]. Outdoor pig farming allows pigs to express a wider range of behaviours in a semi-natural environment [13]. However, outdoor access increases the risk of both endo- and ecto-parasite infection, due to favourable conditions for the development and survival of various stages of parasites in the outdoor environment [14]. Transmission occurs through ingestion of infective eggs, oocytes and larvae. Common parasites in outdoor pigs include protozoa (e.g. *Eimeria* spp., *Isospora suis*, *Balantidium coli* etc.) and helminths (e.g. strongyles, *Ascaris suum*, *Trichuris suis*, *Metastrongylus* spp.) [15]. Several studies report the prevalence of a range of parasite species in outdoor reared pigs in Europe. Deslart et al. [16] assessed 70 alternative farms in France and reported 79% of the farms having coccidia and 47%, 16% and 36% of farms having *Oesophagostomum* spp., *Ascaris suum* and *Trichuris suis* respectively. There are also differences in the prevalence of parasites in different age groups of pigs [17]. Carstensten et al. [18] assessed 9 organic pig herds in Denmark and found *Ascaris suum*,* Oesophagostomum* spp. and *Trichuris suis* among weaners, fatteners and sows with varying levels of prevalence. Băieş et al. [17] examined 960 free-range pigs in Romania and observed that the pigs had *Eimeria* spp., *Balantidium coli*, *Ascaris suum*, *Oesophagostomum* spp., *S. ransomi* and *Cryptosporidium* spp. Furthermore, the abundance of parasites, and hence the potential infective pool in the paddock fluctuated with the seasons, indicating parasite sensitivity to temperature changes [17, 19].

Outdoor pig farming is not an established industry in the island of Ireland; instead, most outdoor farms operate on a small – scale, backyard basis rather than at a commercial level. Menant et al. [20] reported an average of 7 sows/gilts, one boar, 18 grower pigs and 17 piglets in these small scale production units at any one time, which were generally managed by two people. The main breeds were Duroc, Oxford Sandy and Black, Tamworth, and Gloucester old spot pigs. Pigs were reared in agro-forestry systems, on pasture or in a mixture of both. Considering the small-scale nature of the industry, it is poorly characterised, and literature on GI parasites is scarce. In order to address this knowledge gap, the objectives of this study were to:

1. Identify and evaluate the prevalence of GI parasites in outdoor reared pigs on selected farms.
2. Determine the effect, if any, of season, and age on the parasite faecal egg counts of outdoor pigs.
3. Assess the effect of anthelmintic treatment and paddock rotation on the parasite egg burden of outdoor pigs.

## Materials and methods

### Farms and animals

Twenty farmers were selected from 57 respondents to the survey described by Menant et al. [20], and who expressed an interest in volunteering for the study. Farms were located in 12 out of 32 counties across the island of Ireland (Antrim − 1, Clare − 1, Cork − 3, Down − 1, Galway − 1, Laois − 3, Louth − 1, Sligo − 1, Tipperary − 1, Waterford − 3, Wexford − 1, Wicklow − 3). Each farm was visited twice: once between February and May 2023, plus December 2023 (winter/spring, mean ± sd: temperature: 11 ± 4.3 °C, relative humidity: 75.7 ± 11.2%, wind: 1.7 m/s) and once between July and October 2023 (summer/autumn, mean ± sd: temperature: 14.9 ± 4.3 °C, relative humidity: 78.6 ± 8.6%, wind: 0.9 m/s). The first visit encompassed 19 farms, with 17 farms visited during the second phase (Table 1). All of the assessed farms had an electrified fenced outdoor area where the pigs had access to soil, and 6 operated to an organic standard (Table 1). The average size of the paddocks was 21.6 ha (range 0.02–323 ha) with an average of 32 (range 7–128 pigs) pigs per paddock. Information on animal demographics (breed, age) and management practices such as anthelmintic usage (yes/no) and paddock rotation (yes/no) were also collected (Table 1). Anthelmintic use and paddock rotation practices were recorded through semi-structured interviews with farmers. Farmers reported the life stage at which animals were administered anthelmintics by a veterinarian and the frequency of paddock rotation. Farms were classified as using anthelmintics (“Yes”) if they had administered anthelmintics within the previous year and as not using anthelmintics (“No”) if they had never administered them. The frequency of anthelmintic use varied among farms, ranging from administration after weaning, three times per year, to only when deemed necessary. Among the farms that practiced paddock rotation, one farm rotated weekly, two farms rotated every two weeks, three farms rotated monthly, one farm rotated every six weeks, two farms rotated bimonthly, three farms rotated every four months, and one farm rotated annually. Pigs were raised on natural or sown pastures. In the agroforestry systems, the underfoot surface was a litter layer. Pigs older than 3 months were selected for inclusion, while lactating sows and piglets under 3 months old were excluded due to practical issues with data collection and the aggressive nature of the lactating sow. Animals were stratified into two age categories: fatteners (comprising growing and finishing pigs under 8 months old) and sows and boars (encompassing sows and boars aged 9 months to 4 years, Table 1).

### Sample collection

A maximum of 4 faecal samples were collected in each paddock in which pigs were present. The pigs were observed until defecating freely, samples were promptly collected after voiding, then placed into 100 ml plastic cups, and stored in a cool box under chilled conditions (0–4 °C). Each sample within a paddock was combined in equal proportions to create a composite paddock-level sample. Only faecal samples with solid nature were collected. In total, 65 composite paddock level samples were collected over the two visits (Table 1), 37 samples in the first visit, and 28 samples during the second visit. These samples were maintained under chilled conditions during transportation and stored in a cold room at 4 °C until the coprological analysis was conducted. All samples were analysed within 21 days after collection.

**Table 1:** Characteristics of the farms visited and number of paddocks assessed

Farm no	Breeds	Organic system	Visit		Age		Anthelmintic use		Paddock rotation	
			1^st^	2^nd^	Fatteners	Sows/boars	Yes	No	Yes	No
1	Duroc, GOS, OSB, Tamworth	No	√	√	√	√	√		√	
2	Duroc, OSB	Yes	√	√	√	√		√	√	
3	Duroc, Hampshire, Large Black, OSB	Yes	√	√	√	√		√	√	
4	Berkshire, OSB, Tamworth	Yes	√	√	√	√		√	√	
5	Duroc, OSB	No	√	√	√	√		√	√	
6	British saddleback, Duroc, GOS, Hampshire	No	√	√	√	√		√		√
7	OSB, OSB cross breeds	No	√	√	√	√	√		√	
8	OSB	No	√	√	√	√	√		√	
9	OSB	No	√	√	√	×	√			√
10	Tamworth, Vietnamese pot bellied	Yes	√	√	√	√		√	√	
11	Tamworth	Yes	√	√	√	√		√	√	
12	Duroc, OSB, Tamworth	No	√	√	√	√	√		√	
13	Large Black, Middle White, Tamworth	No	√	√	√	√	√		√	
14	Duroc, British Saddleback	Yes	√	×	×	√		√		√
15	Kune Kune, Idaho pasture pigs	No	√	×	√	√		√	√	
16	Duroc, GOS, Landrace	No	√	×	√	√		√	√	
17	Mangalista	No	√	√	√	√		√	√	
18	Berkshire, Duroc, Large White	No	√	√	√	×		√	√	
19	Landrace, Large White	No	√	√	√	√	√		√	
20	Duroc, OSB	No	×	√	√	√		√	√	
	**Number of paddocks**	**65**	**37**	**28**	29	36	27	38	59	6

*GOS* Gloucester Old Spot, *OSB* Oxford Sandy and Black

#### Coprological analysis

A modified McMaster floatation method described by Taylor et al. [15] was employed for the analysis of faecal egg count (FEC), employing NaCl as the floating solution. Initially, 3 g of faecal matter from the composite sample was placed into a mortar and homogenized with 42 ml of chilled water (0–4 °C). The resulting homogenized sample was sieved through a 150 μm sieve, and the filtrate was transferred to a 15 ml centrifugal tube, which was subsequently centrifuged at 1500 rpm for 2 min. The supernatant was removed after centrifugation and added saturated NaCl solution. A vortex disrupted the pellet to ensure thorough mixing. The tubes were inverted 8–10 times, and the mixture was pipetted from the middle of the tube and transferred to a two-chambered McMaster slide. Subsequently, the grid of the slide was examined using a microscope (Alphaphot – 2 YS2, Nikon Corporation, Tokyo, Japan) at 10x magnification to detect parasite eggs. Parasite eggs and protozoa cysts were identified based on their morphology [15], and the number of different eggs in the grid per chamber were counted. The technique had a lower limit of 50 eggs/g per sample. Three replicates (slides) were analysed for each faecal sample. The analysis was performed by one trained person. The FEC is displayed as eggs per gram of faeces (eggs/g) and calculated using the following equation:

$$\begin{aligned}Faecal\:egg\:count=&(egg\:count\:in\:chamber\:01\\&+egg\:count\:in\:chamber\:02)\times\:50\end{aligned}$$

### Statistical Analysis

Statistical analysis of the data were performed using Rstudio (R version 4.2.1, R core team, 2022).

#### Prevalence of parasites within visits

Prevalence at the farm level was determined considering the presence or absence of parasite eggs or oocysts. A farm was considered infected with a parasite if at least one egg was detected. The prevalence value for a parasite taxon was calculated as a percentage of farms infected from the number of all the farms for both visits separately. Fisher’s exact test was used to analyse the prevalence of a parasite between visits.

#### Effect of the season and age on the faecal egg count

A Generalized Linear Mixed Effect Model, utilizing the glmmTMB package [21] was employed to investigate the impact of season and age on the FEC. To address the zero-inflation observed in the data, we applied a negative binomial distribution with a log link function, along with zero-inflated adjustments. Fixed effects included season and age while paddock nested within farm was considered as a random effect to account for potential clustering effects. The interaction between season and age was considered. Subsequently, a Type III Analysis of Variance (ANOVA from car package; [22]) was conducted to validate the model’s findings. Estimated marginal means were then derived for further interpretation of the results and pairwise comparisons (emmeans package; [23]) between the visits and the age groups were conducted.

#### Effect of using anthelmintics and paddock rotation on the faecal egg count

A similar Generalized Linear Mixed Effect Model was used to analyse the effect of using anthelmintics and paddock rotation on FEC. Anthelmintic usage, paddock rotation, and their interaction were included as fixed effects, while paddock nested within farm was used as a random effect.

## Results

### Parasite taxa

**Fig. 1:**
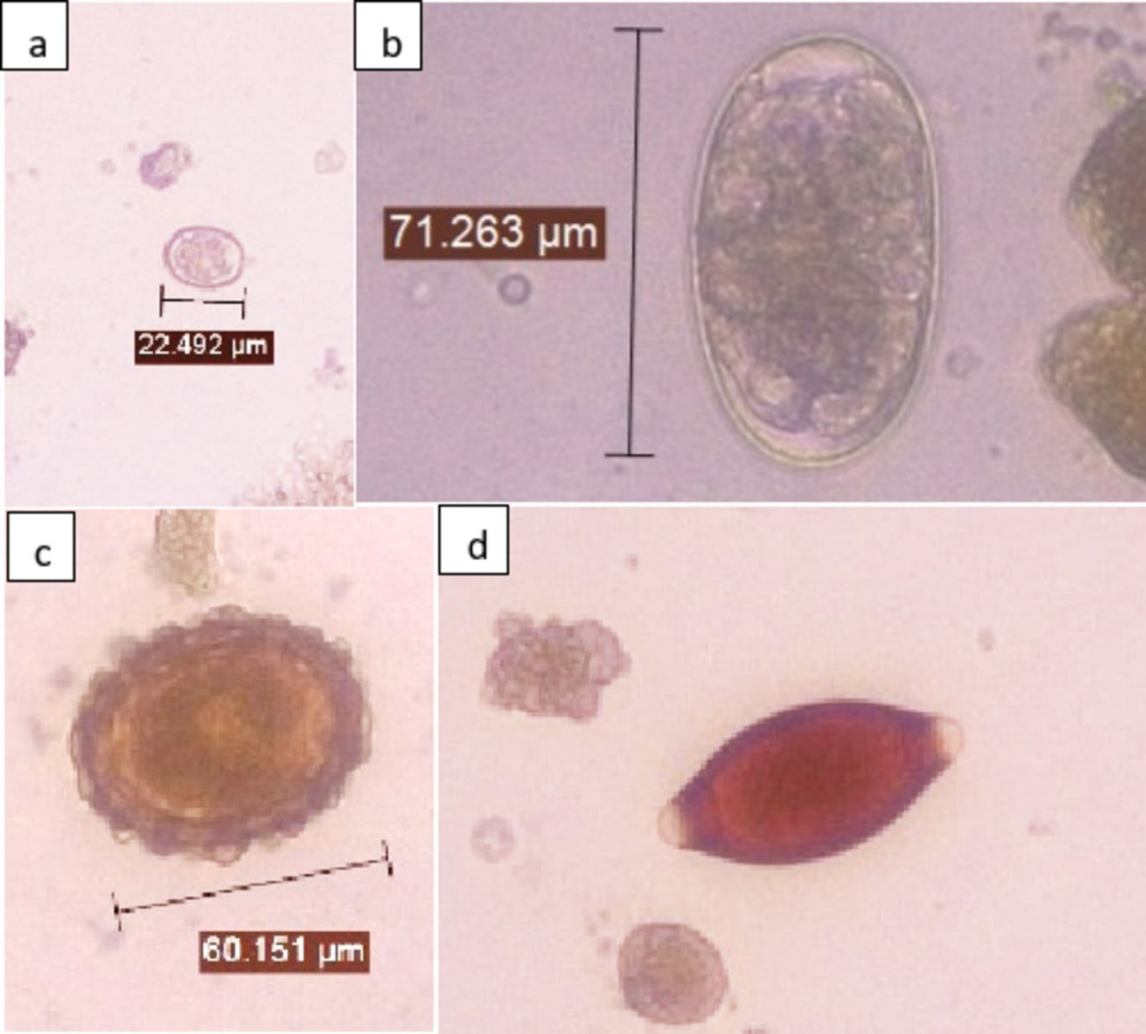
Photographs of *Eimeria/Isospora* spp. oocyst (**a**), strongyle eggs (**b**), *Ascaris suum* egg (**c**) and *Trichuris suis* egg (**d**) identified on bright-field microscope (20x magnification)

We identified four taxa of parasite eggs based on egg morphology (Fig. 1). They were *Eimeria/Isospora* spp., strongyles, *Ascaris suum* and *Trichuris suis*. Strongyles were grouped because several species (*Oesophagostomum* spp., *Hyostrongylus rubidus*, *Trichostrongylus* spp. etc.) have similar morphology making it impossible to identify to species level.

### Prevalence of parasites

Prevalence of *Eimeria/Isospora* spp. and strongyles was high (> 80%) at both visits and *Ascaris suum* prevalence was moderate, at approx. 30%. *T. suis* was found on one farm during the 1 st visit and on two farms on the 2nd visit (Table 2). There was no significant effect of the visit on the prevalence of each parasite (Table 2).

**Table 2:** Number and percentage of farms with the presence and absence of gastro-intestinal parasites during the two visits (Fisher’s exact test to determine the significance between farms in two visits)

Season	1^st^ visit (19 farms)		2^nd^ visit (17 farms)		*P*-value
	Present	Absent	Present	Absent	
*Eimeria/Isospora *spp.	16 (84%)	3 (16%)	16 (94%)	1 (6%)	N.S
Strongyles	17 (89%)	2 (11%)	14 (82%)	3 (18%)	N.S.
*Ascaris suum*	6 (32%)	13 (68%)	5 (29%)	12 (71%)	N.S.
*Trichuris suis*	1 (5%)	18(95%)	2 (12%)	15 (88%)	N.S.

*N.S* Not Significant

### Effect of season and age on faecal egg count

**Fig. 2:**
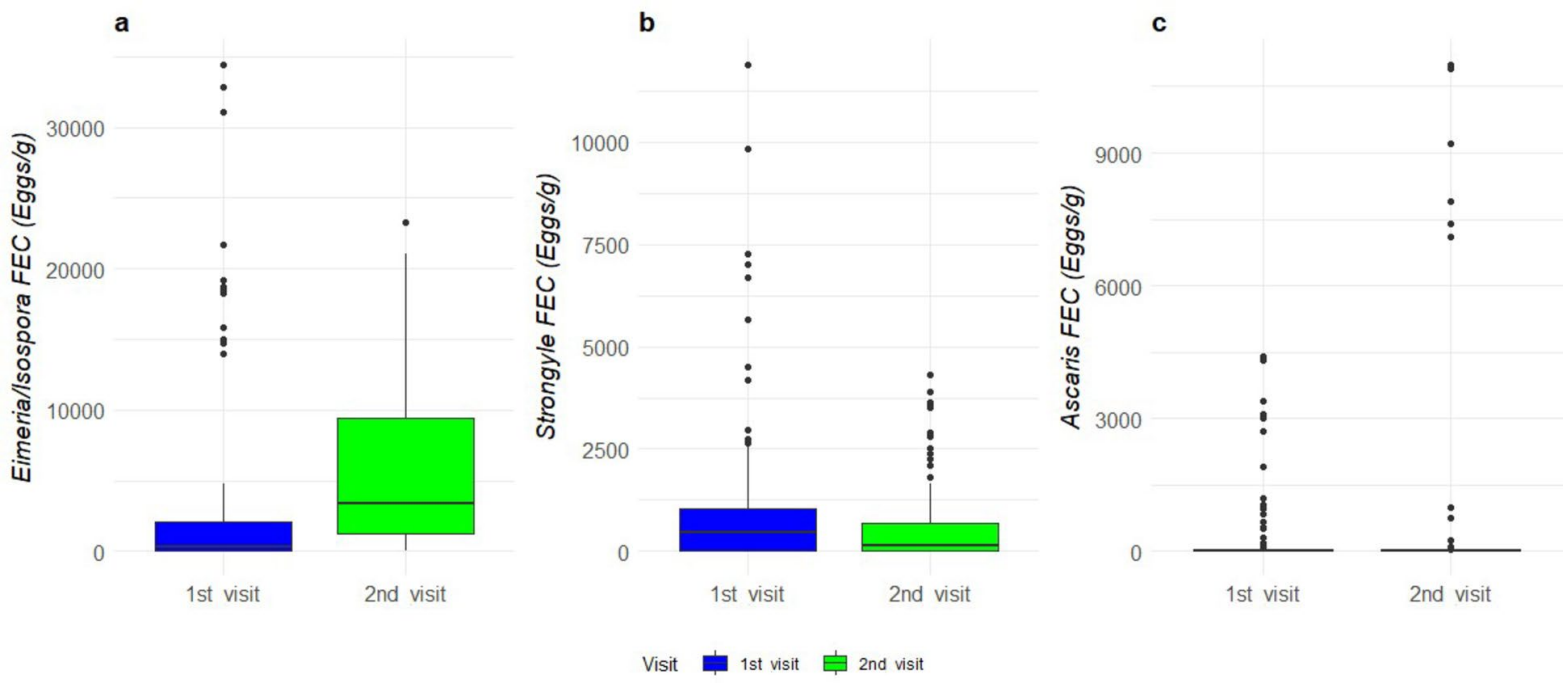
Median and quartiles of (**a**) *Eimeria/Isospora* spp., (**b**) strongyles and (**c**) *A. suum* faecal egg counts (FEC) during the 1^st^ (blue, winter/spring) and 2^nd^ visit (green, summer/autumn)

**Fig. 3:**
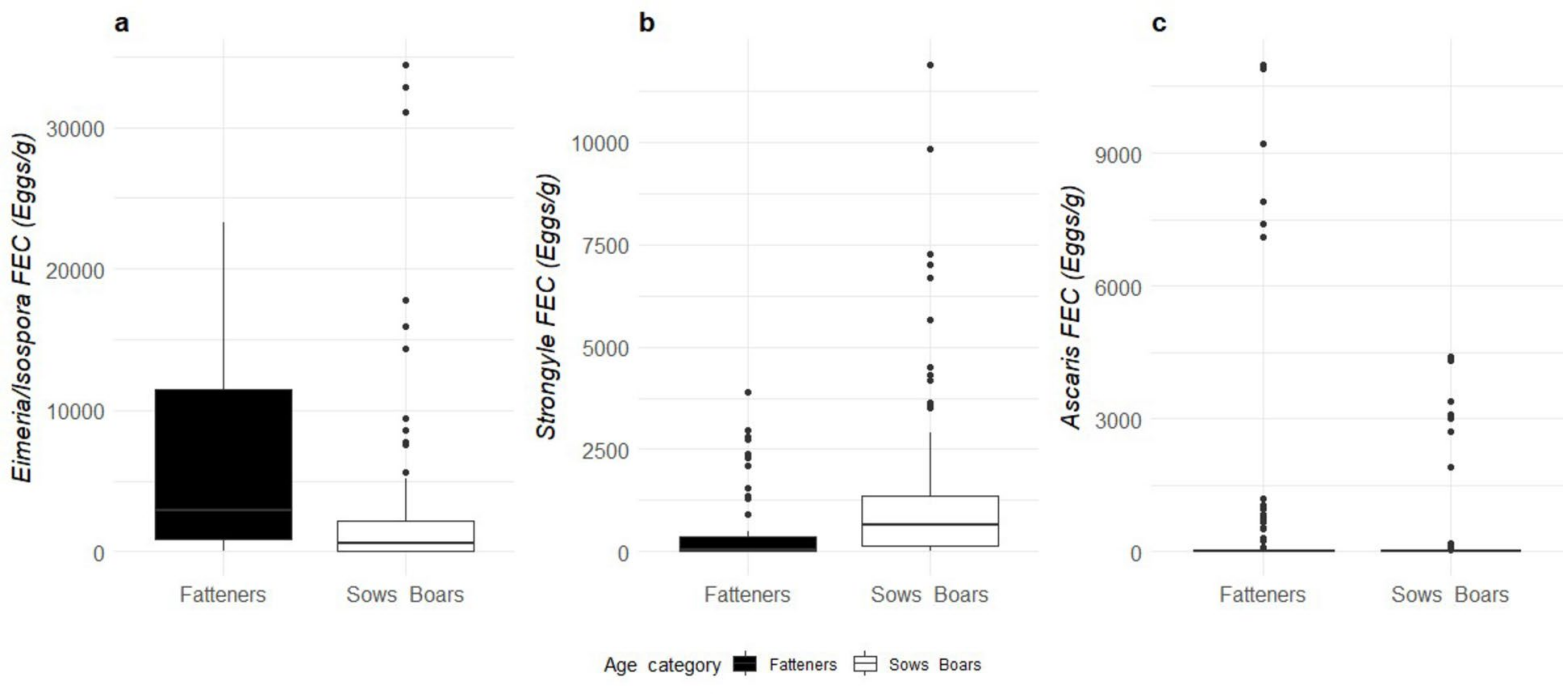
Median and quartiles of (**a**) *Eimeria/Isospora* spp., (**b**) strongyles and (**c**) *A. suum* faecal egg counts in relation to fatteners (black) and sows/boars (white)

*Eimeria/Isospora* spp. count was higher during the 2nd visit compared to the 1 st visit (Estimated marginal means ± sd; 1 st visit – 476 ± 297 vs. 2nd visit – 1527 ± 950 eggs/g; *P* < 0.001, Fig. 2a). Furthermore, fattener pigs had higher *Eimeria/Isospora* spp. FEC than sows and boars (fatteners – 1175 ± 745 vs. sows/boars – 619 ± 386 eggs/g; *P* < 0.05, Fig. 3a). Strongyle FEC was higher in the 1st visit than in the 2nd visit (1 st visit – 370 ± 198 vs. 2nd visit – 141 ± 77 eggs/g; *P* < 0.01, Fig. 2b) and sows and boars had higher strongyle FEC than fatteners (fatteners – 125 ± 71 vs. sows and boars – 419 ± 223 eggs/g; *P* < 0.01, Fig. 3b). There was no interactive effect between season and age in the analysis. There was no effect of season or age for *Ascaris suum* FEC (Figs. 2c and 3c). Since *Trichuris suis* was found on only two farms it was not statistically analysed.

### Effect of using anthelmintics and paddock rotation on faecal egg count

**Fig. 4:**
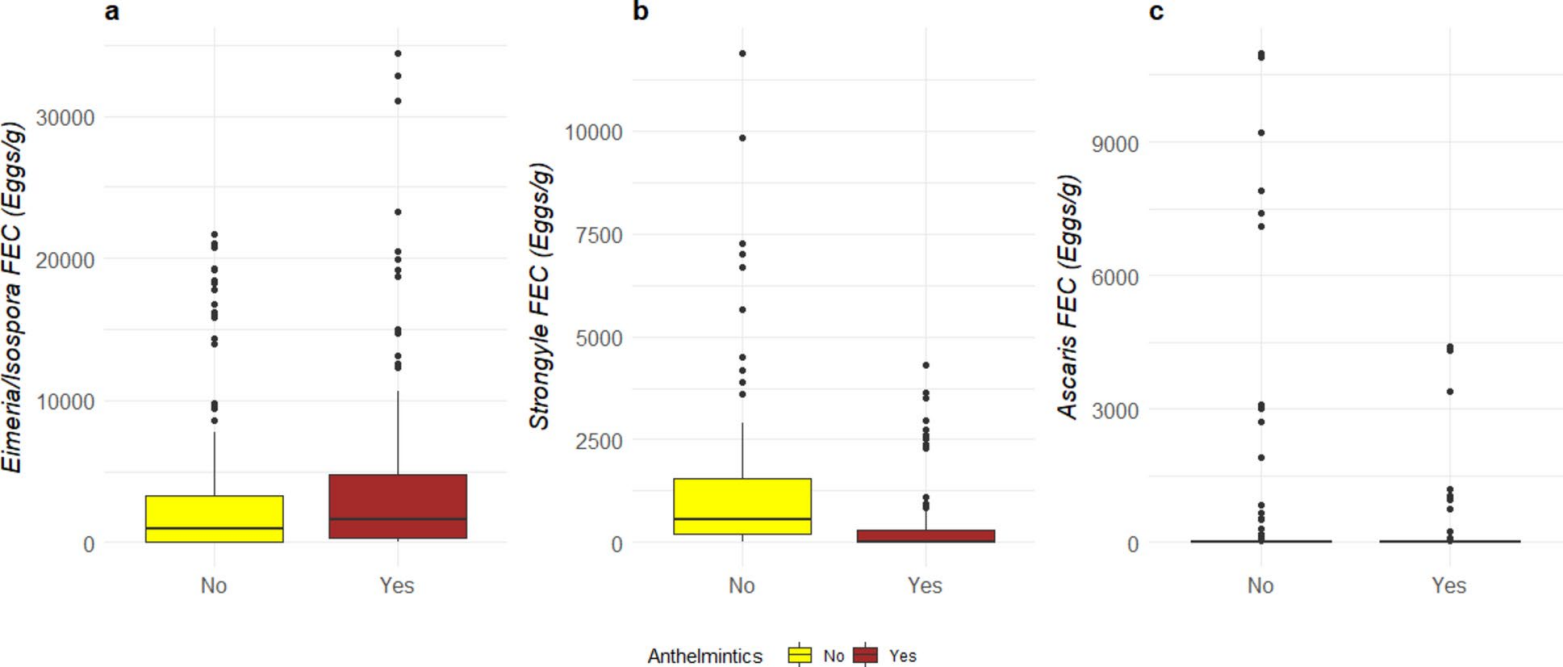
Median and quartiles of (**a**) *Eimeria/Isospora* spp., (**b**) strongyles and (**c**) *A. suum* in relation to anthelmintic usage (Yes – Brown, No – Yellow)

The farms that used anthelmintics had lower strongyle FEC in both seasons (Yes: 48.2 ± 37.6 eggs/g, No: 668.3 ± 381.7 eggs/g; *P* < 0.01, Fig. 4a). Neither *Ascaris suum* nor *Eimeria/Isospora* spp. FEC levels were affected by anthelmintic use (Fig. 4b, c).

**Fig. 5:**
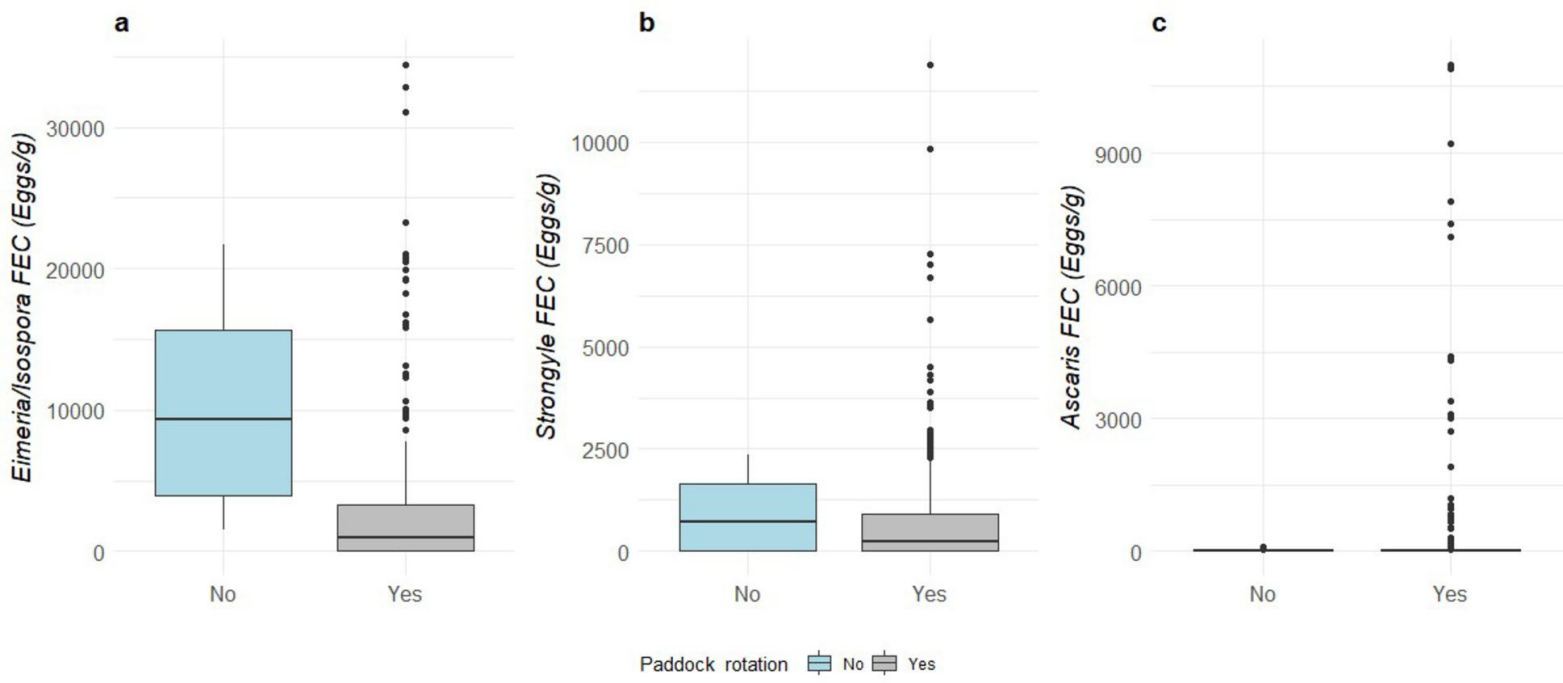
Median and quartiles of (**a**) *Eimeria/Isospora* spp., (**b**) strongyles and (**c**) *A. suum* in relation to paddock rotation (Yes – grey, No – light blue)

There was no effect of paddock rotation on *Eimeria/Isospora* spp., strongyles or on *Ascaris* FEC (Fig. 5). There was no interaction between paddock rotation and use of anthelmintics. However the use of anthelmintics in the farms influenced paddock rotation in the additive model and the farms which practiced paddock rotation had lower levels of *Eimeria/Isospora* spp. FEC when anthelmintics was administered (Anthelmintics yes, paddock rotation yes – 1629 ± 651 eggs/g vs. Anthelmintics yes, paddock rotation no – 7188 ± 5646 eggs/g; *P* = 0.05).

## Discussion

This study provides the first data on the prevalence of a range of taxa of GI parasites found in pigs produced outdoors on the island of Ireland. The only other work on pig GI parasites conducted in Ireland reported on liver damage caused by *Ascaris suum* in intensively produced pigs [24]. In the present study, season, age of the pig, and the anthelmintic usage had significant effects on FEC related to both *Eimeria/Isospora* spp. and strongyles and influenced paddock rotation with *Eimeria/Isospora* spp. counts.

### Prevalence of the parasites

A high proportion (> 80%) of the farms were infected by *Eimeria/Isospora* spp. and strongyles at both visits. Similarly, a Romanian study found a high prevalence of *Eimeria* spp. during both summer (80%) and winter (77.5%) and with a somewhat lower prevalence for strongyles (*Oesophagostomum* spp.); 27.5% during summer and 37.5% during winter [17]. A survey of 101 pig farms in Western Australia recorded evidence of nematode parasites in 79% of farms with 65% positive for O*esophagostomum* spp. and 55% positive for coccidia across all the ages of pigs [25].

The oocysts of *Eimeria* spp. show great resistance to environmental conditions, making high prevalence and year round presence possible in pigs [26]. The high prevalence of strongyles could also be due to year round shedding of the eggs by the adult worms. Thus, even though the eggs have a low resistance to climatic extremes, compared with other helminth eggs, the possibility of the hatched larvae being ingested is present throughout the year, even while the burden differs.

Prevalence of *Ascaris suum* (30%) affected farms in this study was more or less similar to some other studies conducted in Europe across all farm systems. *Ascaris suum* had a 28.6% prevalence in Poland, from 70 pig farms [10]. A study in Estonia found 31.5% prevalence of *Ascaris suum* in ecological and small pig farms (*n* = 20 farms; [27]). Free-range farms in Netherlands had a 50% prevalence of *Ascaris suum* (*n* = 27 farms; [28]) and Rodrigues Da Costa et al. [24] found, 30% of the commercial pig farms in Ireland had pigs with milk spots, which occurs due to *Ascaris suum*. The observed similar prevalence (30%) suggesting that outdoor pigs may have no greater a risk of *A. suum* infection than those raised indoors although the different method of estimation should be considered (i.e. milk spots vs. egg counts).

In the present research, very few farms were affected by *Trichuris suis* as evidenced by the low presence of faecal eggs (one in the 1 st visit, (5%), and 2 in the 2nd visit, (12%)). Other studies recorded a much higher proportion of farms (ranging from 21.4 to 37.5%) affected by this parasite [10, 28]. However, the relatively quick expulsion of adults, and thus egg-laying individuals, by the host reduces the value of FEC alone in assessing prevalence of this helminth species among hosts [6].

### Effect of season and age on the faecal egg count

In intensive systems, Symeonidou et al. [29] found that *Crisoisospora suis*, a coccidia species similar to *Eimeria* spp., tended to have a lower burden of oocysts in winter compared to spring. Another investigation by Băieş et al. [17] found a different parasitic load of *Eimeria* spp. between the seasons winter/spring (higher) and summer/autumn (lower). In this study, the highest mean number of *Eimeria/Isospora* spp. oocysts was recorded in the 2nd visit, which was during late summer/autumn. *Eimeria* oocysts can die when the temperature is colder (− 20 °C) or hotter (38 °C), as in the case of *Eimeria bovis*, a coccidia which infects cows [30]. Mild summer temperatures, such as those that occur in Ireland (mean temperatures between 14 and 15 °C; [31, 32]) result in favourable conditions for the *Eimeria* oocysts to become infective, causing the higher FEC during the summer and autumn month periods.

Infection from strongyles occurs from the L3 infective larval stage. Eggs are passed through faeces, hatch, and develop infective larvae, which are ingested by the pigs [33]. Harsh, cold, winters and dry, hot, summers have detrimental effects on *Oesophagostomum* spp. eggs, leading to higher mortality rates [34]. According to Nansen and Roepstorff [31], the lower temperature limit for the *Oesophagostomum* spp. is ~10 °C and the eggs deposited during the winter do not survive into the spring. Rose and Small [35] found that transmission of both *Oesophagostomum* spp. and *Hyostrongylus rubidus* could not take place during winter in British conditions. Even though a higher temperature average (> 7 °C) was observed during the 1 st visit, Irish winter air temperature averages are at 4–7 °C [32]. The highest mean faecal egg counts (FEC) of strongyle-type nematodes were observed during the 1 st visit, conducted in late winter/spring. The strongyle lifecycle within the pig spans approximately 4–6 weeks [15]. In outdoor environments, most eggs and free-living larvae do not survive the winter months due to harsh conditions. This results in a reduced larval load available for ingestion by pigs, leading to fewer mature adult worms and consequently lower FEC during the summer/autumn months. However, Ireland’s mild summers, compared to other European countries, may not significantly affect the survival of eggs and free-living larvae. This could contribute to the higher FEC observed during the winter/spring period, as larvae surviving through the summer are ingested by pigs and develop into adult worms capable of producing eggs during these colder months.

During this study, the faecal egg counts of *Ascaris suum* and *Trichuris suis* did not differ between the two visits, although they were present on only 30% of the farms visited. The eggs of these parasites are more resistant to environmental conditions, which could explain the consistency of their counts relative to those of strongyles [36].

Several studies mention significant differences in the FEC between pigs of different ages in terms of *Eimeria* spp. and strongyles [16, 17, 37]. In the present analysis, fattener pigs had higher mean FEC for *Eimeria/Isospora* spp. while the sows and boars had higher mean FEC for strongyles. Older animals (sows and boars) have stronger immunity towards *Eimeria* spp. due to frequent contact with *Eimeria* spp. and other coccidia [16]. This could explain the lower FEC in these older animals. In contrast, the patterns for strongyles such as *Oesophagostomum spp.* and *Hyostrongylus rubidus* were of higher parasite intensity with increasing host age [18]. *Oesophagostomum* spp. has low immunogenicity, resulting in almost all larvae surviving into maturity and living inside the pig for 2–4 months [8] thus the higher strongyles FEC observed during our study in these older animals.

In contrast, *Ascaris suum* and *Trichuris suis* have high immunogenicity and a large majority of larvae are expelled from the small intestine, leaving a small number of mature worms inside the animal [8]. The results of this study did not indicate an age effect on FEC; however, in several studies from other European countries the prevalence and intensity of *A. suum* and *T. suis* differed across age groups (intensity lowered with the age of the animal) [18, 27].

### Effect of using anthelmintics and paddock rotation on the FEC

Ireland, being a member of the European Union, has a list of approved anthelmintics for use in pigs. Anthelmintics classes include Benzimidazoles, Levamisole and Macrocyclic lactones [38]. Mooney et al. [39] listed a total of 40 anthelmintic compounds approved for use in Ireland, encompassing a range of drugs applicable to various livestock species, including pigs. In our study only 7 of the 20 farms administered anthelmintics to their pigs. As the results indicated, the farms using anthelmintics had significantly lower strongyle counts. Indeed, the use of anthelmintics on strongyle species such as *Oesophagostomum* spp. is highly effective, even though anthelmintic resistance is an emerging issue [6]. Although other studies show high efficacy (in some studies > 90%) of anthelmintics on *Ascaris suum*, regardless of the class used [6, 40], the present study did not find an effect of anthelmintic use on *Ascaris suum* FEC. However, only 3 of the 7 farms that used anthelmintics had *Ascaris suum* and this may have influenced the sensitivity of the analysis. A survey of 101 pig herds in Western Australia found that 80% of pig herds treated with anthelmintics still showed evidence of parasitic infection, suggesting that a single-dose treatment may not be the most effective deworming strategy [41].

Paddock rotation is an important management method to control parasites. Anthelmintics alone are not effective in the long -run, as parasites and eggs can survive in the soil or be re-introduced by other reservoirs [42] so re-infection is inevitable. This could explain why in this study, the FEC of *Eimeria/Isospora* spp. in the farms that practised paddock rotation was low even when the use of anthelmintics was taken into account.

## Conclusions

This analysis observed seasonal variation in the FEC of *Eimeria/Isospora* spp. and strongyles, with *Eimeria/Isospora* spp. levels higher in the winter/spring, and strongyle levels higher in the summer/autumn. Grower and fattener pigs had higher *Eimeria/Isospora* spp. counts while sows and boars had higher strongyle counts, indicating different parasite community dynamics in different age groups. Anthelmintic usage seems to be effective against strongyles, and paddock rotation tended to reduce *Eimeria/Isospora* spp. counts in the presence of anthelmintics. There is a lack of data on GI parasites in Irish pigs, particularly those kept outdoors, and this study sheds light on the factors that could affect their prevalence.

## Data Availability

No datasets were generated or analysed during the current study.

## Abbreviations

GI: Gastro–intestinal
FEC: Faecal egg count
